# Myocardial bridging presenting as myocardial ischaemia induced cardiac arrest: a case report

**DOI:** 10.1186/s12872-021-01975-x

**Published:** 2021-04-14

**Authors:** Young-Jae Ki

**Affiliations:** Departments of Internal Medicine, College of Medicine, School of Medicine, Chosun University, 588 Seosuk-dong, Dong-gu, Gwangju, 61453 Republic of Korea

**Keywords:** Myocardial bridging, Cardiac arrest, Myocardial ischaemia

## Abstract

**Background:**

Myocardial bridging is a congenital anomaly defined as a segment of epicardial coronary arteries running through the myocardium. Various complications related to myocardial bridging have been reported, but at present, cardiac arrest has rarely been reported.

**Case presentation:**

We report one case of a patient who was successfully resuscitated from ventricular fibrillation cardiac arrest and was diagnosed with myocardial bridging accompanied by myocardial ischaemia. A 50-year-old woman who had been resuscitated from cardiac arrest transferred to our institution for evaluation and management of out-of-hospital cardiac arrest. We confirmed the diagnosis of significant myocardial bridging with evident myocardial ischaemia by coronary angiography, resting echocardiography and heart MRI. Vasospasm was thought to be a trigger factor judging from the transient ST elevation on electrocardiography. In addition, the finding of septal buckling was detected for the first time throughout the whole cardiac cycle by resting echocardiography in MB.

**Conclusion:**

We report a rare case of survival after out-of-hospital cardiac arrest that might be caused by significant myocardial bridging-induced myocardial ischaemia, which was objectively confirmed by echocardiography and heart MRI. Although myocardial bridging is often overlooked as an aetiology for sudden cardiac death, this case highlights the importance of expanding the differential diagnosis to myocardial bridging in the work-up for the cause of sudden cardiac death.

## Background

Myocardial bridging (MB) is a form of congenital anomaly featuring a major coronary artery that takes an intramuscular course. One meta-analysis reported that MB prevalence varies depending on the diagnostic methods. The prevalence of MB was highest in autopsy studies at 42%, followed by 22% in computed tomography (CT) and 6% in coronary angiography (CAG) studies [1]. This finding means that MB is considerably underdiagnosed since most patients are asymptomatic. Although MB has been generally considered a benign disease, some serious complications have been reported, including myocardial infarction (MI), takotsubo syndrome, coronary spasm and sudden cardiac death (SCD) [2–5]. Of these complications, SCD has been reported intermittently in the literature, and there is still a long debate over the causality [6]. In this respect, we report a unique case of a young woman resuscitated from ventricular fibrillation (VF) cardiac arrest who was diagnosed with MB accompanied by evident myocardial ischaemia as the cause.

## Case presentation

**Fig. 1:**
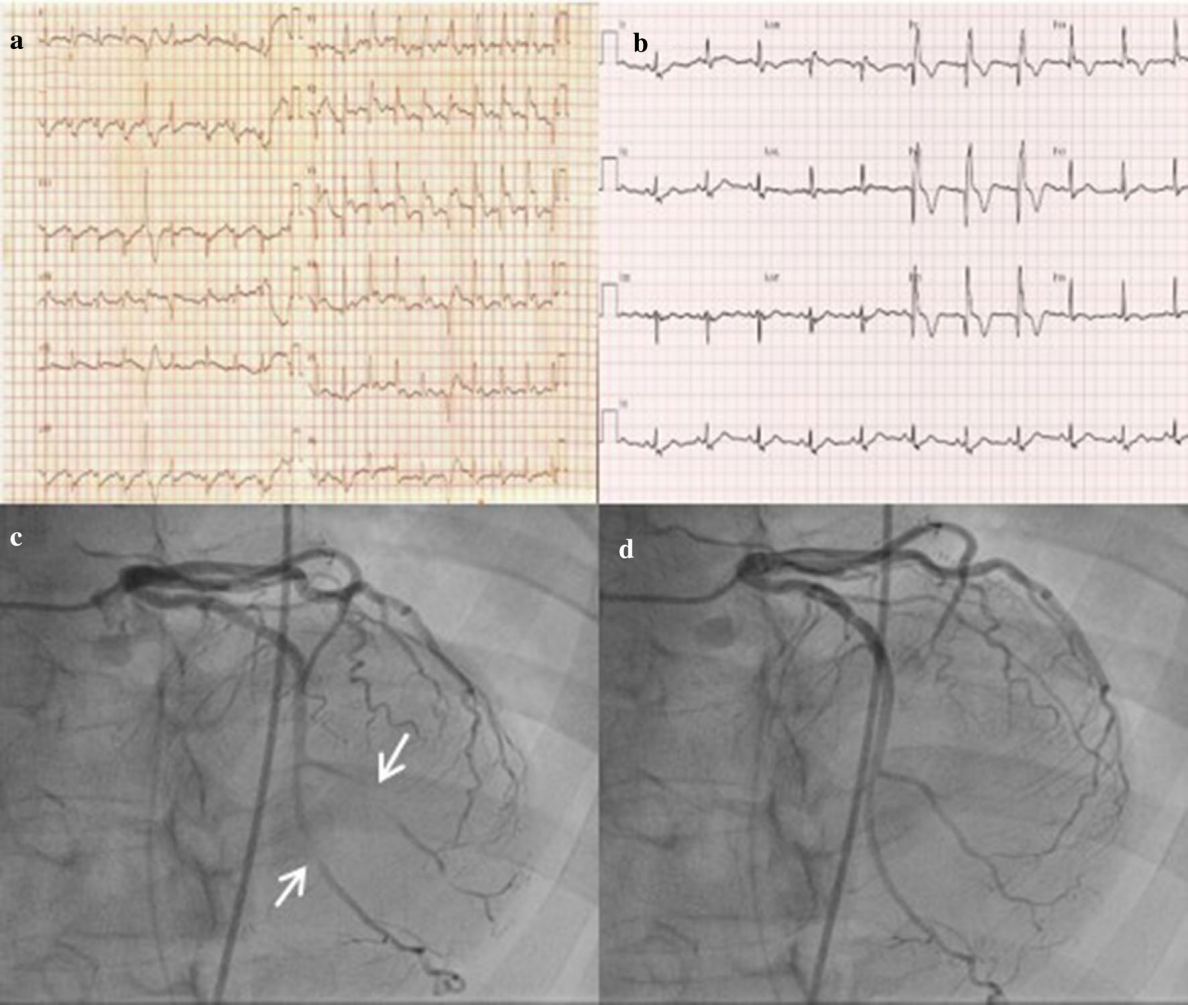
The electrocardiogram (ECG) performed in the local emergency department showed a normal sinus rhythm with a right bundle branch block (RBBB) with ST elevation and Q-waves over V1–V4 (**a**). ECG performed at our institution showed complete resolution of ST elevation on V1–V4 (**b**). Coronary angiography showed significant myocardial bridging in the distal segment of the left anterior descending coronary artery and diagonal branch with compression up to 100% stenosis during systole (arrow) (**c**, **d**)

A 50-year-old woman with no previous comorbidity and no family history transferred to our institution for further evaluation of VF cardiac arrest. Two hours prior to this admission, she collapsed suddenly with no alarming symptoms and suffered a witnessed cardiac arrest while having lunch. Cardiopulmonary resuscitation (CPR) was performed by a witness immediately. Shortly thereafter, she transferred to the nearby local emergency department. She was subsequently intubated for airway protection, and her initial rhythm was documented as VF. Therefore, 10 min of CPR and 5 rounds of defibrillation were performed, and return of spontaneous circulation (ROSC) was achieved. Her post-defibrillation electrocardiogram (ECG) showed a right bundle branch block (RBBB) with ST elevation and Q-waves over V1–V4 (Fig. 1a). Upon our clinical examination, she had high blood pressure of 160/80 mmHg and was otherwise haemodynamically stable. Her mental status was stupor, and non-contrast head CT showed no evidence of haemorrhage. Auscultation of both lungs revealed a clear breath sound, and no heart murmur was audible. Laboratory evaluation showed an increased white blood cell count (14,980/μL, normal = 4000–10,800/μL), prohormone of brain natriuretic peptide (proBNP) (998.1 pg/mL, normal = 0–270 pg/mL), troponin-I (0.054 ng/mL, normal = 0–0.016 ng/mL) and D-dimer (3725 ng/mL, normal = 0–225 ng/mL). The creatinine kinase-myocardial band (CK-MB) (3.69 ng/mL, 0–6.22 ng/mL) and potassium (3.8 mEq/L, normal = 3.5–5.0 mEq/L) were within normal limits.

An ECG performed in our emergency room showed an RBBB with a deep Q-wave over V1–V4 and resolution of ST elevation (Fig. 1b). Given that her post-defibrillation ECG showed ST elevation on the precordial lead, we planned to perform urgent coronary angiography (CAG) and spasm provocation tests under the suspicion of MI or variant angina. There was no significant stenosis of either coronary artery with anomalous origin of the right coronary artery (RCA). Significant MB on the distal segment of the left anterior descending artery (LAD) and diagonal branch was observed (Fig. 1c, d). Considering the transient precordial lead ST elevation on ECG, we performed a spasm provocation test. After administration of ergonovine (total dose 50 µq) into the left coronary artery (LCA), no spasm and no ECG changes were observed, and a provocation test on RCA failed because cannulation of anomalous origin RCA was difficult.

Shortly after CAG, she underwent hypothermia treatment for 72 h, after which she gradually regained consciousness without any neurological deficits. On day 4 of hospitalization, she was successfully weaned off the ventilator with good neurological recovery. Subsequently, careful evaluation was performed to identify the cause of VF cardiac arrest.

**Fig. 2:**
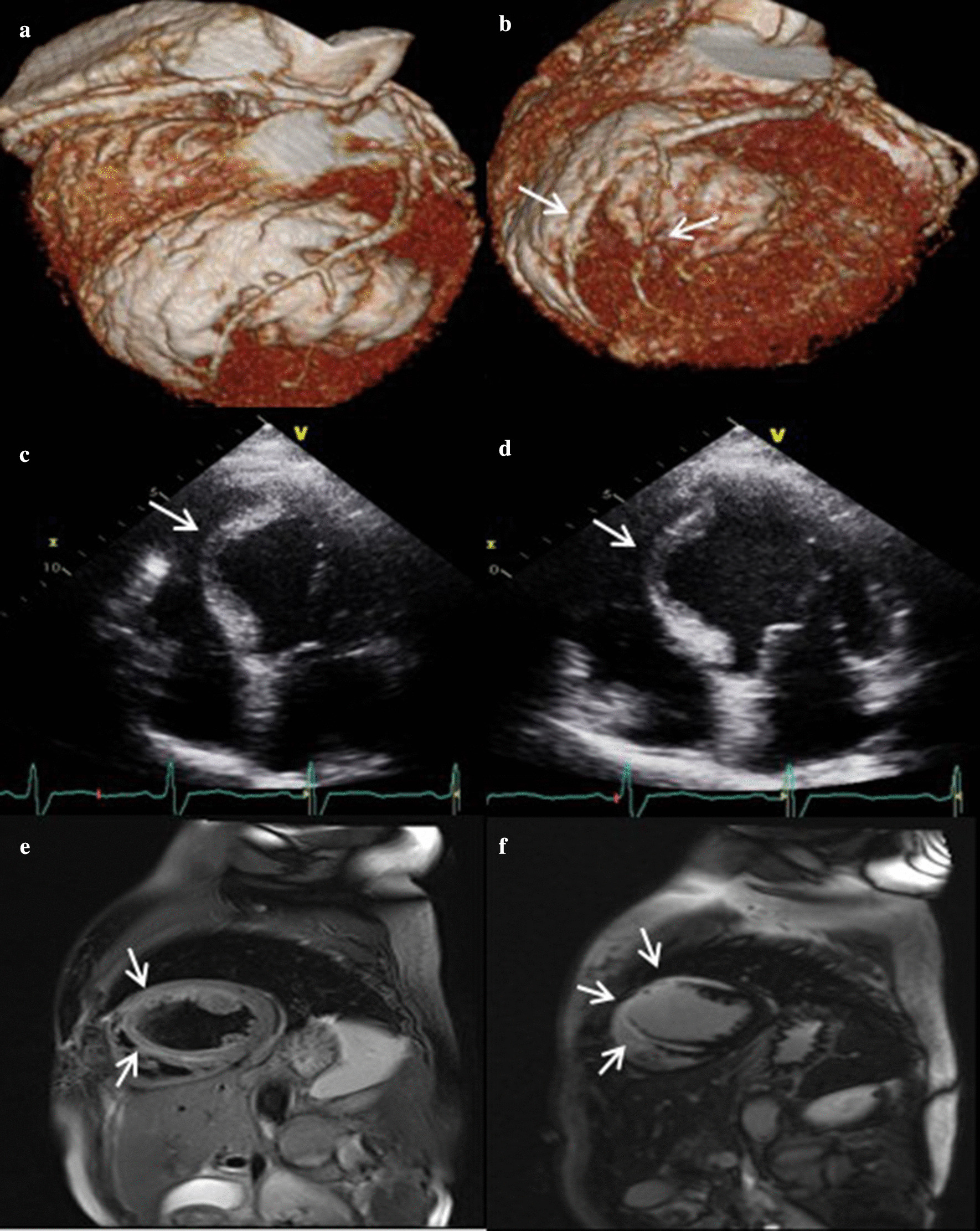
3D volume rendered reconstruction of the heart and coronary arteries showed high take off of the right coronary artery (**a**) and distal segment of the left anterior descending coronary artery and diagonal branch with a superficial intra-myocardial course (arrow) (**b**). Resting echocardiography showed focal septal wall motion abnormalities called septal buckling (arrow) at end systole (**c**) and early diastole (**d**). T2-weighted heart MRI image showed myocardial wall thinning and subendocardial myocardial oedema (arrow) in the mid-ventricular to apical anterior and septal regions (**e**). Late gadolinium enhancement imaging showed a high signal intensity in the same region (**f**)

Given the increased d-dimer level, we performed coronary CT angiogram (CCTA). We could exclude pulmonary thromboembolism and confirm that there was no stenosis on anomalous origin RCA. We also checked for an intra-myocardial course of the distal LAD and diagonal branch, and the lengths of tunnelled segments were 31 mm and 30.7 mm, respectively (Fig. 2a, b).

Transthoracic echocardiography demonstrated focal mid-septal wall motion abnormalities with an ejection fraction of 52% (Fig. 2c, d). Based on this finding of ischaemia, we asked her if she had complained of shortness of breath or chest discomfort before this event, and she said that there had been no specific symptom during her exercise or working out. For further evaluation of ischaemia and elucidation of other causes of cardiac arrest, we performed heart magnetic resonance imaging (MRI). This procedure revealed myocardial wall thinning and subendocardial oedema in the mid ventricular to apical anterior and septal regions, and myocardial late gadolinium enhancement imaging revealed myocardial scarring with > 50–75% transmurality in the same region (Fig. 2e, f). In light of these results, we concluded that MB might be the cause of myocardial ischaemia and VF cardiac arrest and that vasospasm might play a role as a trigger factor.

The patient was discharged on day 25 of hospitalization after full resolution of her presenting symptoms with no neurologic sequelae. She was on a calcium channel blocker and angiotensin receptor blocker and was asymptomatic over the 1-year follow-up.

## Discussion and conclusions

To date, individual case reports have described MB pertaining to SCD. Among them, several studies confirmed MB as a cause of SCD through an autopsy or using intracoronary Doppler flow velocity measurement [6–10], while another study revealed the cause of SCD as an MB through the exclusion diagnosis process [3]. However, none of the studies clearly presented evidence of heart MRI and echocardiography-based myocardial ischaemia as playing a role in SCD in MB patients. To our knowledge, this is the first case report that explains the causal relationship between MB-induced myocardial ischaemia and VF cardiac arrest.

MB is an anatomical variant featuring coronary artery tunnelling through the myocardium. MB has a relatively benign prognosis; however, fatal cardiac events sometimes occur, as in our case. There has been a question of whether MB is an incidental finding or a cause of myocardial ischaemia. Given that 70% to 95% of coronary blood flow occurs during diastole, myocardial ischaemia from MB, which induces systolic compression of the coronary artery, has been thought to be uncommon [11]. However, there has been growing evidence that MB is closely linked to myocardial ischaemia. First, studies have demonstrated that compression of the coronary artery persists in the diastolic phase, which impedes early diastolic myocardial perfusion [12–14]. Exercise and emotional stress induce tachycardia, and sympathetic overactivation further shortens diastolic filling time and lengthens MB contraction, both of which can lead to myocardial ischaemia and angina symptoms. In addition, atherosclerosis can develop on proximal entrance to the MB segment as a result of a disturbed blood flow pattern and endothelial injury in the upstream segment [15]. Notably, MB has diverse anatomy depending on the depth and length of encasement [16]. The length of MB induces myocardial ischaemia through haemodynamically significant stenosis on the entrapped segment, and side branches originate from the compression segment [15]. Indeed, a recent meta-analysis revealed that MB is strongly associated with myocardial ischaemia and subsequently increases the risk of major adverse cardiac events or MI [17, 18]. In addition to the aforementioned points, left ventricular diastolic dysfunction, hypertension, left ventricular hypertrophy, coronary vasospasm, and microvascular dysfunction can induce symptoms of myocardial ischaemia in previously asymptomatic patients [16]. In the current case, we considered vasospasm, which was confirmed by transient ST elevation on ECG, to be a trigger factor.

MB can be an independent risk factor for the development of interstitial fibrosis through myocardial ischaemia-induced myocardial damage. This diffuse fibrosis might play a role as an arrhythmogenic risk factor [19, 20]. Based on this evidence, we hypothesize that MB-induced chronic myocardial ischaemia causes structural heart disease, which might be an arrhythmogenic substrate in this patient.

Patients with MB are usually asymptomatic and may be diagnosed incidentally or undiagnosed. In practice, there was a report that MB was found in 23% of 331 patients in the intravascular ultrasound (IVUS) group compared to only 3% in the CAG group [21]. Recently, CCTA has been in the spotlight as a non-invasive method with high accuracy that is just as good as IVUS [22]. Echocardiography is also a widely used non-invasive method with good accessibility, and several findings, such as myocardial stunning and stress-induced cardiomyopathy, have been reported in MB patients [23, 24]. Studies utilizing stress echocardiography (SE) have reported a novel finding of transient focal septal wall motion abnormality with apical sparing called septal buckling during end systole to early diastole. Septal buckling was associated with a documented MB by IVUS and a decreased diastolic functional flow reserve (dFFR). Moreover, this finding was explained by local ischaemia rather than distal ischaemia, which was caused by a decrease in the corresponding septal branch perfusion pressure during systolic compression of the MB segment [25]. There was an additional report that the presence of septal buckling on SE is a precise predictor of an MB in patients with angina who presented non-obstructive coronary artery disease (CAD) by CAG [26]. In this case, we also confirmed septal buckling on resting echocardiography (RE) throughout the whole cardiac cycle. In light of this result, we can hypothesize that if MB is severe enough to induce persistent myocardial ischaemia, septal buckling could be confirmed during the whole cardiac cycle by RE, as in our case. However, the exact role of echocardiography needs to be further studied until it is routinely used as a diagnostic test in MB patients.

Our case report includes three limitations. First, we could not completely rule out the possibility of coronary spasms. As previously known, chronic mechanical stress on MB segments could lead to focal endothelial dysfunction and vasospasm [5]. Although a spasm provocation test could not be performed on the RCA, considering the fact that there was a transient ST elevation on the precordial lead and the provocation test was done on the LCA with negative results, provocation failure on the RCA did not seem to be a problem for future treatment decisions. However, we are still considering that vasospasm on the MB segment on the LAD might play an important role as a trigger factor. Second, we did not perform additional intracoronary imaging, such as IVUS, intracoronary Doppler or intracoronary pressure wire assessment, because of the emergency situation at that time. There has been growing evidence of the aforementioned imaging tests in symptomatic MB patients for confirming diagnoses and assessing the haemodynamic significance of stenosis of MB [2]. Instead of that invasive method, we confirmed myocardial ischaemia by non-invasive methods, such as RE and heart MRI, after the patient had fully recovered from the emergency situation. Third, we also needed to manage the patient more aggressively. Although we recommended some preventive and management options during her hospitalization, the patient seemed reluctant because of personal problems. We plan to continue recommending additional study and preventive management every time she comes to see the doctor.

Here, we report the unique case of survival after VF out-of-hospital cardiac arrest that might have been caused by significant MB-induced myocardial ischaemia, which was confirmed objectively by RE and heart MRI. In addition, we report for the first time the characteristic findings of echocardiography called septal buckling throughout the whole cardiac cycle by RE, not SE.

MB, due to its rarity, is often overlooked as an aetiology for SCD. Based on these case reports, we highlight the importance of expanding the differential diagnosis to MB in the work-up for the cause of SCD.

## Data Availability

All data generated or analysed during this study are included in this published article.

## Abbreviations

MB: Myocardial bridging
CT: Computed tomography
CAG: Coronary angiography
MI: Myocardial infarction
SCD: Sudden cardiac death
VF: Ventricular fibrillation
CPR: Cardiopulmonary resuscitation
ROSC: Return of spontaneous circulation
ECG: Electrocardiogram
RBBB: Right bundle branch block
CAG: Coronary angiography
RCA: Right coronary artery
LAD: Left anterior descending artery
CCTA: Coronary CT angiography
MRI: Magnetic resonance imaging
IVUS: Intravascular ultrasound
SE: Stress echocardiography
RE: Resting echocardiography
